# Interaction between ingested nutrients and gut endocrine cells in patients with irritable bowel syndrome (Review)

**DOI:** 10.3892/ijmm.2014.1811

**Published:** 2014-06-17

**Authors:** MAGDY EL-SALHY, ODD HELGE GILJA, DORIS GUNDERSEN, JAN G. HATLEBAKK, TRYGVE HAUSKEN

**Affiliations:** 1 Section of Gastroenterology, Department of Medicine, Stord Helse-Fonna Hospital, University of Bergen, Bergen, Norway; 2 Section of Gastroenterology, Department of Clinical Medicine, University of Bergen, Bergen, Norway; 3 National Centre for Ultrasound in Gastroenterology, Department of Medicine, Haukeland University Hospital, Bergen, Norway; 4 Department of Research, Helse-Fonna, Haugesund, Norway

**Keywords:** allergy, cholecystokinin, diet, endocrine cells, enteric nervous system, gluten, minerals, polypeptide YY, probiotics, serotonin

## Abstract

Several endocrine cell abnormalities have been reported in different segments of the gastrointestinal tract of patients with irritable bowel syndrome (IBS). These cells have specialized microvilli that project into the lumen; they function as sensors for the gut contents and respond to luminal stimuli (mostly ingested nutrients) by releasing hormones into the lamina propria, where they exert their effects via a paracrine/endocrine mode of action. Certain food items trigger the symptoms experienced by IBS patients, including those rich in fermentable oligo-, di- and monosaccharides, and polyols (FODMAPs). In this review, we present the argument that the effects of both FODMAPs and the proportional intake of proteins, fats and carbohydrates on IBS symptoms may be caused by an interaction with the gut endocrine cells. Since the gut hormones control and regulate gastrointestinal motility and sensation, this interaction may be responsible for abnormal gastrointestinal motility and the visceral hypersensitivity observed in these patients. There is no consistent evidence that IBS patients suffer from food allergy. The role of gluten intolerance in the development of IBS symptoms in these patients remains a matter of controversy. Individual guidance on food management, which includes restrictions in the intake of FODMAP-rich foods and testing diets with different proportions of proteins, fats and carbohydrates has been found to reduce the symptoms, improve the quality of life, and make the habitual diet of IBS patients more healthy.

## Introduction

Irritable bowel syndrome (IBS) is a chronic functional gastrointestinal disorder with a worldwide prevalence of 10–20% (1–15). The diagnosis of IBS is based mainly on the assessment of the symptoms, which are abdominal discomfort/pain, altered bowel habits and abdominal bloating/distension (1,4). Patients with IBS can be subdivided into four subtypes according to the Rome III criteria and based on the stool pattern: diarrhea-predominant (IBS-D), constipation-predominant (IBS-C), mixed diarrhea and constipation (IBS-M) and unclassified IBS (U-IBS) (16,17).

IBS is usually diagnosed in younger patients (i.e., <50 years of age) and is more common in women than in men (3–6,8,9,11, 12,14,15,18,19). Although IBS is not known to be associated with the development of serious disease or with excess mortality, it considerably reduces the quality of life of patients (1,19–21). In addition to the increased morbidity caused by IBS, this condition represents an economic burden to society as a result of the overconsumption of healthcare resources by and low productivity of IBS patients (22).

IBS patients often associate their symptoms with specific food items, such as milk and milk products, wheat products, caffeine, certain meats, cabbage, onion, peas/beans, hot spices, fried foods and smoked foodstuffs (23–25). However, surveys of the diets of IBS patients have failed to detect any differences in diet composition between IBS patients and the community as regards the intake of energy, carbohydrates, proteins and fats (26–32). However, a study on food intolerance and IBS found that 62% of the subjects had either limited or excluded food items from their daily intake, and 12% of these subjects had made such drastic changes in their diet that nutritional deficiencies could be foreseen in the long term (33).

Certain studies have found IBS patients to be intolerant to various alcoholic beverages and generally have a low alcohol consumption (23,29). However, other studies found that the alcohol intake in patients with IBS was the same as or higher than that in the background population (30,31). The common belief among IBS patients is that lactose is the main cause of their symptoms, and consequently, they often reduce their intake of milk and milk products (29,31,34,35). Milk and other dairy products are the most important dietary source of calcium, vitamin B2 (riboflavin) and phosphorus in the Western world (36). Thus, while IBS patients consume more products that are alternatives to milk, such as soy, rice and oat milk, they have a low daily intake of calcium, vitamin B2 and phosphorus (29).

IBS patients have a lower consumption of foods known to be rich in fermentable oligo-, di- and monosaccharides, and polyols (FODMAPs), such as spaghetti, pasta, rice, millet, couscous and buns than healthy controls (29). Moreover, IBS patients have lower consumptions of certain vegetables (raw vegetables, raw broccoli, paprika, onion, leeks, garlic, cabbage, tomatoes, mushrooms and green beans) (29). On the other hand, they consume more FODMAP-rich fruits and vegetables, such as grapes, pears, peaches, peas, mango, plums and melon (29).

The importance of dietary factors and the associations between diet and symptoms in IBS have been discussed in the literature (23,37–41). The aim of this review was to shed light on the possible interaction between dietary intake and gut hormones, and the importance of diet management in reducing the symptoms and improving the quality of life of IBS patients.

## 2. The role of diet in IBS

The effect of diet on IBS symptoms may be attributed to the interaction between poorly absorbed carbohydrates/fiber and the intestinal bacterial flora, or between ingested nutrients and the gut neuroendocrine system, and food allergy or intolerance.

### Interaction between poorly absorbed carbohydrates/fiber and the intestinal bacterial flora

Certain short-chain carbohydrates (FODMAPs) are poorly absorbed, resulting in a significant proportion of them reaching the distal small bowel and colon (42,43), where they provide a substrate for bacterial fermentation. This results in the production of gas, with the consequent distension of the large intestine and increased intraluminal pressure. FODMAPs include fructose, lactose, sugar alcohols (sorbitol, maltitol, mannitol, xylitol and ismalt), fructans and galactans. Fructose and lactose are present in apples, pears, watermelon, honey, fruit juices, dried fruits, as well as milk and milk products. Polyols are used in low-calorie food products. Galactans and fructans are present in wheat, rye, garlic, onions, legumes, cabbage, artichokes, leeks, asparagus, lentils, inulin, soy, Brussels sprouts and broccoli (39,40,44). A low intake of FODMAPs has been found to reduce the gastrointestinal symptoms in patients with IBS (42,43,45,46).

Increasing the intake of dietary fiber is a standard recommendation for patients with IBS (47). However, in clinical practice, increased fiber intake in these patients has been shown to increase the symptoms of abdominal pain, bloating and distension. The examination of the effects of fiber intake on IBS symptoms has revealed that increased fiber intake does not improve symptoms compared with a placebo or a low-fiber diet (47). However, it has been reported that the intake of soluble fiber is effective in improving overall IBS symptoms relative to consuming insoluble fiber (47–50).

The effects of FODMAPs and fiber on IBS symptoms are strongly associated with the intestinal flora. The dominance of *Clostridium* spp. in the intestinal flora, which break down FODMAPs and fiber, results in gas production, with a consequent increase in the distension of the large intestine, causing abdominal discomfort or pain. Food supplements with beneficial bacteria, such as *Lactobacillus* spp. and *Bifidobacterium* spp. would result in a greater tolerance to both FODMAPs and fiber, since these bacteria do not produce gas on fermenting carbohydrates. It has been reported that the intestinal flora of IBS patients comprise fewer *Lactobacillus* spp. and *Bifidobacterium* spp. than the flora of healthy individuals (51,52).

### Interaction between ingested nutrients and the gut neuroendocrine system

**Figure 1:**
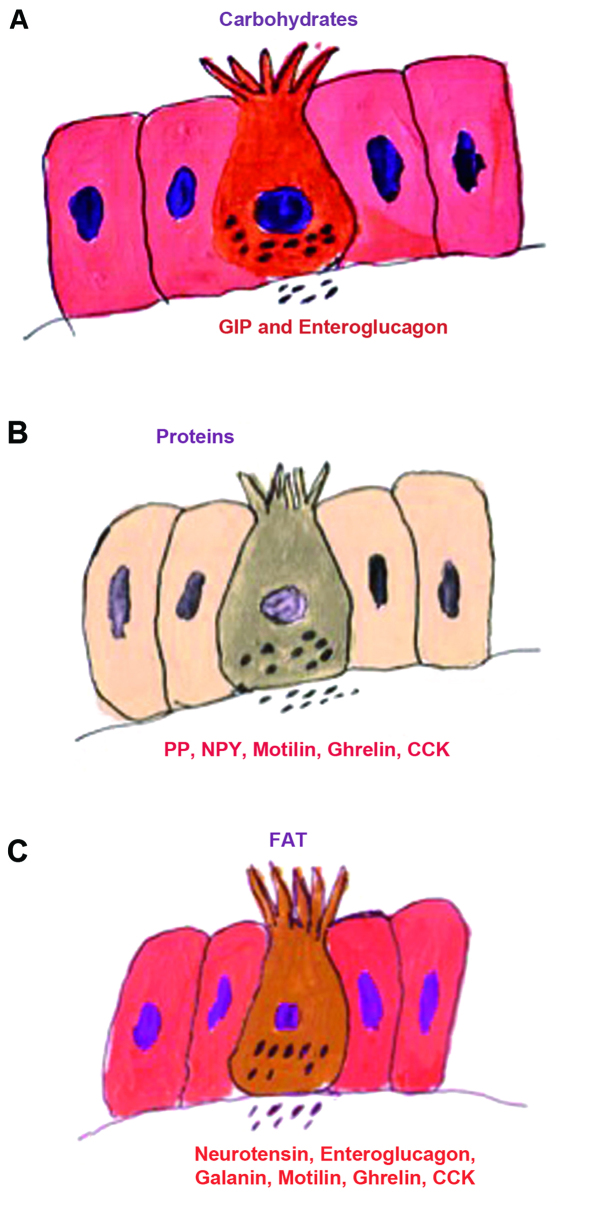
The gut hormones released into the interstitial fluid of the lamina propria in response to intraluminal nutrient content vary according to the proportions of (A) carbohydrates, (B) proteins and (C) fats. These hormones may act in an endocrine/paracrine manner or as neurotransmitters/neuromodulators of neurons in the ENS.

The gut endocrine cells are spread between the epithelial cells of the mucosa facing the gut lumen (1,53). They are present in all the segments of the gastrointestinal tract apart from the esophagus (1). There are several different populations of gut endocrine cells (22,32,53–55); the distribution, functions and modes of action of the most important types have been reported previously (22,32,53,56–68). Some of the different endocrine cell types are located only in specific areas of the gut, while others are found throughout the gut (53–55). Thus, serotonin- and somatostatin-secreting cells are found throughout the gastrointestinal tract, while those producing ghrelin and gastrin are found in the stomach; those producing secretin, cholecystokinin (CCK), gastric inhibitory peptide (GIP) and motilin are found in the upper small intestine, and those producing polypeptide YY (PYY), pancreatic polypeptide (PP) and enteroglucagon are located in the lower small intestine and large intestine (53–55). These cells have specialized microvilli that project into the lumen and function as sensors for the gut contents (mostly for nutrients), and respond to luminal stimuli by releasing their hormones into the lamina propria (Fig. 1) (69–81). The gut intraluminal contents of carbohydrates, proteins and fats triggers the release of the different signaling substances (i.e., hormones) from the gut endocrine cells (1,53). These signaling substances may exert their actions locally on nearby structures (paracrine mode) or by entering the circulating blood and reaching distant targets (endocrine mode) (82). The gut endocrine cells interact and integrate with each other, and with the enteric nervous system (ENS) and the afferent and efferent nerve fibers of the autonomic nervous system and the central nervous system (CNS) (22,53,59,83). In doing so, they regulate several functions of the gastrointestinal tract, including visceral sensation, motility, secretion, absorption, local immune defense and food intake (22,53–55,83).

**Figure 2:**
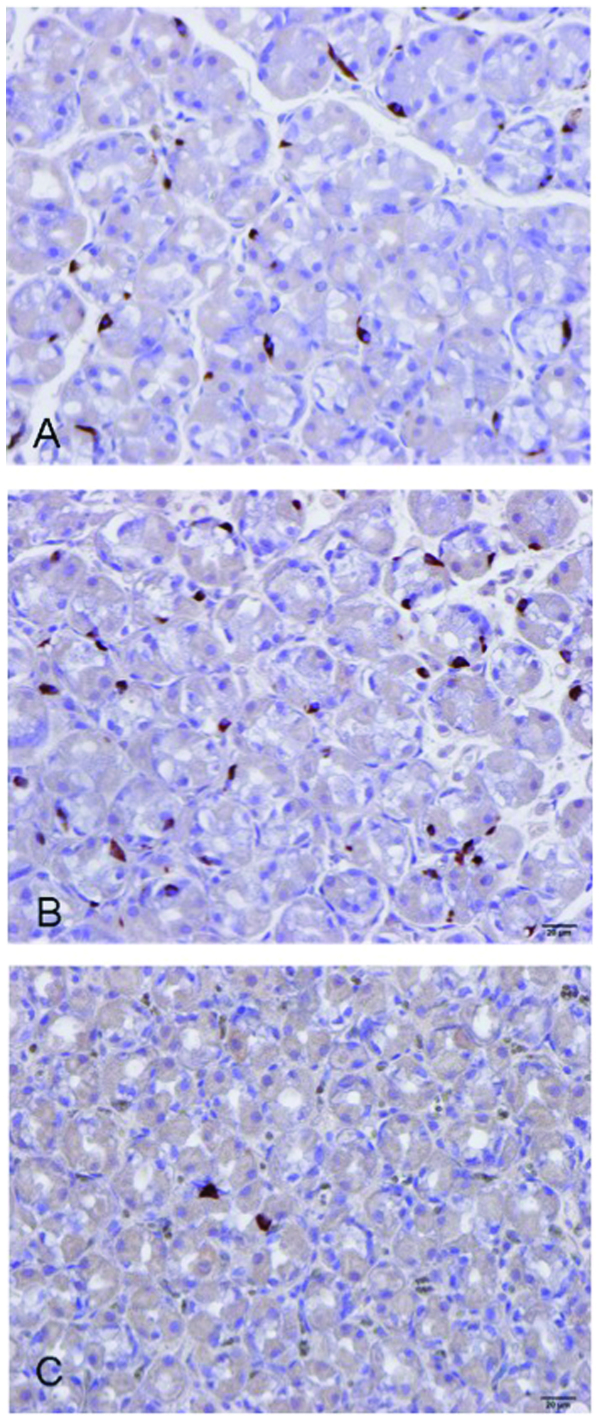
Ghrelin in the oxyntic mucosa of (A) a healthy subject, (B) a patient with diarrhea-predominant IBS (IBS-D), and (C) a patient with constipation-predominant IBS (IBS-C).

**Figure 3:**
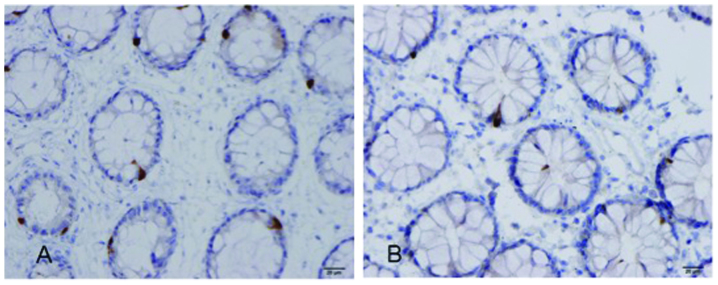
Polypeptide YY (PYY)-immunoreactive cells in the colon of (A) a healthy subject and (B) a patient with irritable bowel syndrome (IBS).

Several abnormalities in the gut endocrine cells have been described in IBS patients (84–100), as summarized in Table I and illustrated in Figs. 2 and 3. The etiology of these abnormalities in sporadic (non-specific) IBS patients can be genetically inherited and/or caused by environmental factors. A genetic etiology is supported by the familial aggregation of IBS and the results of twin studies (101–111). Alternatively, endocrine cells have a rapid turnover, and it is possible that factors related to luminal content, such as diet or bacterial flora can provoke an increase or decrease in the endocrine cell population (54,55). In post-infectious IBS (PI-IBS), the abnormalities in gut endocrine cells may be the result of endocrine/immune interactions (i.e., the endocrine/immune axis), which are in turn caused by low-grade inflammation following gastroenteritis in predisposed individuals (112,113).

As indicated in Table I, gastrointestinal hormone release is triggered by the intraluminal contents of nutrients; thus, the release of ghrelin, CCK and PYY is triggered by proteins and fat, and ghrelin release is suppressed by the presence of carbohydrates. Consequently, while a diet that is poor in fat, proteins and carbohydrates would aggravate the symptoms in patients with IBS-D, a diet containing low levels of fat and proteins and high levels of carbohydrates would worsen the symptoms in patients with IBS-C. In patients with PI-IBS, the symptoms would be worsened by food rich in proteins and fat.

In IBS patients, the gut endocrine cells may be responsible for the abdominal pain/discomfort resulting from the aforementioned gas production and consequent increase in intraluminal pressure and large-intestinal distension following the breakdown of FODMAPs and fibers by the intestinal bacterial flora. An increase in the intraluminal pressure would possibly result in the release of serotonin and substance P into the interstitial fluid. Serotonin activates the submucosal sensory branch of the ENS, which conveys the sensation to the CNS, possibly causing the sensation of abdominal pain/discomfort (114,115). Furthermore, serotonin controls gastrointestinal motility and chloride secretion via interneurons and motor neurons, which may result in disturbances in both motility and gastrointestinal secretion (114,115).

### Food allergy or intolerance

There is neither consistent evidence for an allergic response nor documented evidence for intolerance to a specific food in IBS (1,116–122). Although a food allergy mediated by mucosal mechanisms has been suggested for IBS (123,124), these mechanisms may play a role in only a subset of patients who may have atopy or PI-IBS (1,123,125). Different classes of antibodies (IgG) have been implicated in food-related allergies in IBS (126, 127). The results of studies on this subject are controversial, possibly sicne the tests used are not sufficiently sensitive or specific (31,116,117,123,124,128–133).

The association between IBS and celiac disease (CD) has drawn much attention of late. The breadth of the spectrum of symptoms in IBS means that there is the potential for overlap with CD symptomatologies. Thus, patients with CD presenting with relatively vague abdominal symptoms can be diagnosed as having IBS (39,40). Furthermore, the symptoms of both IBS and CD patients are triggered by the ingestion of wheat products. The reported prevalence of CD in IBS varies between 0.04 and 4.7% (134–144). It has been suggested that IBS patients with wheat intolerance and who carry the genotype associated with CD (HLA DQ2 or DR3), but do not have typical serological markers or changes in small-intestine histology exhibit other immunological evidence of gluten reactivity and response to a gluten-free diet (145).

The augmentation of IBS symptoms by the ingestion of wheat products has been attributed to the content of the sugar polymers, fructans and galactans (38,146). In clinical practice, some IBS patients describe a reduction in symptoms upon eating a gluten-free diet. Although this has been dismissed by clinicians as a placebo effect, there is emerging new data regarding non-celiac gluten sensitivity (147). The existence of non-celiac gluten intolerance has been demonstrated by a double-blinded, randomized, placebo-controlled rechallenge trial (148). However, the diets of the subjects in that study excluded wheat products, which contain gluten, as well as fructans and galactans. A recent placebo-controlled, cross-over study found no evidence of the specific effects of gluten in non-celiac gluten sensitivity (149). Thus, the role of gluten intolerance in IBS has yet to be clarified, and further studies are required.

## 3. Diet management in IBS

It is clear that IBS patients need guidance on diet management. Providing IBS patients with diet guidance has been found to reduce symptoms and to improve their quality of life (29,150). Furthermore, this guidance leads IBS patients to consume a more adequate diet in terms of the levels of vitamins and minerals, and makes them aware of all FODMAP-rich foods, the consumption of which they should either avoid or reduce. They also consume foods supplemented with *Lactobacillus* spp. and *Bifidobacterium* spp., which increase their tolerance to FOODMAPs (29).

Diet guidance should be individualized, since IBS patients have different tolerances to various FODMAP-rich foods, possibly due to differences in their intestinal flora. The aim of diet guidance should be to provide information about FODMAPs and their role in the symptoms of individual patients, and to instruct them to avoid such foods. Moreover, the effects of the proportional intakes of protein, fats and carbohydrates on their symptoms should be examined. In clinical practice, we have found that reducing the carbohydrate or fat intake and increasing the protein intake improves the symptoms in certain patients. In addition, IBS patients should be encouraged to consume foods that are supplemented with *Lactobacillus* spp. and *Bifidobacterium* spp. Other lifestyle factors, such as regular exercise and regular intake of probiotics, may augment the effect of diet management (151).

## 4. Conclusion

Diet triggers symptoms in IBS patients, possibly as a result of interactions with the gut endocrine cells, which are defective in IBS patients. The effects of the food content of FODMAPs and fiber on IBS symptoms are possibly mediated through gut endocrine cells. FODMAPs in the diet increase the osmotic pressure and provide a substrate for bacteria fermentation and gas production in the large intestine, resulting in abdominal distension. The increase in intestinal pressure may cause the release of serotonin and substance P, which in turn may result in the sensation of abdominal discomfort or pain. The protein, fat and carbohydrate content of ingested foods determine the amount and type of gut hormones released, which will in turn regulate and control gastrointestinal motility and sensation, that have been reported to be abnormal in IBS patients (152–179). Although it is possible that IBS patients suffer from gluten intolerance, further studies are required to confirm this before any definitive conclusions can be drawn. Guidance on diet management, including individually tailored restrictions of FODMAP-rich foods and the testing of protein-, fat- and carbohydrate-rich/poor diets reduce IBS symptoms and accordingly improve the overall management of the health of IBS patients.

## Figures and Tables

**Table I:** Summary of the abnormalities in the endocrine cell densities in different segments of the gastrointestinal tract of IBS patients, and the factors responsible for the release of gut hormones and the functions of these hormones.

Gut segment	Endocrine cell type	Released by	Functions	IBS-D	IBS-C
Stomach	Ghrelin	Protein and fat ingestion. Suppressed by carbohydrate ingestion.	Increases gastric and intestinal motility, and stimulates appetite and food intake.	High	Low
	Serotonin	Adrenaline, acetylcholine, acidification, and increased intraluminal pressure.	Activates the submucosal sensory branch of the ENS, inhibits gastric emptying, stimulates colonic motility, and accelerates small- and large-intestinal transit.	Normal	High
	Gastrin	Intraluminal peptides, amino acids, calcium, amines, low pH, and prostaglandins. Release inhibited by somatostatin.	Stimulates gastric acid secretion and histamine release, and stimulates contraction of the LES and antrum.	High	High
	Somatostatin	Meal and acidification of the stomach.	Inhibits gut exocrine and neuroendocrine secretion, and inhibits intestinal contraction.	Low	Low
Small intestine					
Duodenum	CCK	Intraluminal protein and fat.	Stimulates pancreatic exocrine secretion and growth, regulates food intake, inhibits gastric emptying, and stimulates gallbladder contraction and intestinal motility.	Low	Normal
	Secretin	Acidification of the intestinal contents.	Stimulates pancreatic bicarbonate and fluid secretion; inhibits gastric emptying; inhibits contractile activity of the small and large intestines.	Low	Normal
	GIP	Intraluminal glucose; amino acids and fat.	Inhibits gastric acid secretion.	Low	Low
	Somatostatin	Intraluminal glucose; amino acids and fat.	Inhibits gastric acid secretion.	Low	Low
Ileum	Serotonin	Intraluminal glucose; amino acids and fat.	Inhibits gastric acid secretion.	Low	Low
	PYY	Protein- and fat-rich meals.	Delays gastric emptying, stimulates the absorption of water and electrolytes; major mediator of the ileal brake.	Normal	High
Large intestine					
Colon	Serotonin	Protein- and fat-rich meals.	Delays gastric emptying, stimulates the absorption of water and electrolytes; major mediator of the ileal brake.	Low	Low
	PYY	Protein- and fat-rich meals.	Delays gastric emptying, stimulates the absorption of water and electrolytes; major mediator of the ileal brake.	Low	Low
Rectum	PYY	Protein- and fat-rich meals.	Delays gastric emptying, stimulates the absorption of water and electrolytes; major mediator of the ileal brake.	Low	Low
	Enteroglucagon	Intraluminal carbohydrates and fat.	Inhibits gastric and pancreatic secretion, reduces gastric motility, and has also some incretin effect.	Low	Low
	Somatostatin	Protein- and fat-rich meals.	Delays gastric emptying, stimulates the absorption of water and electrolytes; major mediator of the ileal brake.	High	High

IBS, irritable bowel syndrom; IBS-D, diarrhea-predominant IBS; IBS-C, constipation-predominant IBS; LES, lower esophageal sphincter; GIP, gastric inhibitory peptide.
